# Impact of the Gap Between Social Workers' Work Interaction Frequency With Governments and Clients on Their Burnout in China: Mediating Effects of Role Conflict and Moderating Effects of Non-front-line Work

**DOI:** 10.3389/fpubh.2022.908800

**Published:** 2022-06-03

**Authors:** Jie Wang, Zimin Tan, Jiajun Li, Qiang Wu

**Affiliations:** ^1^Center for Chinese Public Administration Research, Guangzhou Research Centre for Social Security, School of Government, Sun Yat-sen University, Guangzhou, China; ^2^Social and Public Administration School, East China University of Science and Technology, Shanghai, China; ^3^Department of Sociology, Sun Yat-sen University, Guangzhou, China; ^4^School of Public Administration and Policy, Renmin University of China, Beijing, China

**Keywords:** social work, government, burnout, role conflict, role ambiguity, front-line, China

## Abstract

**Background:**

Since the 2000s, local governments have contracted out more and more social services to social work organizations in China. Social workers are thus experiencing the inconsistency between local governments' and clients' demands and the deviation from the professional duty of helping clients, which may result in conflicting and unclear roles in their jobs and further lead to burnout. Based on the Role Stress-burnout Model and the previous theoretical and field-work investigations, this study examined the effects of the government-client work interaction frequency gap on social workers' burnout as well as the mediating effects of role ambiguity and conflict and the moderating effects of the non-front-line work.

**Methods:**

A cross-sectional study of 2,643 front-line social workers and 2,599 supervisors or managers from 56 major cities all over China was conducted. Work burnout was measured by the 22-item three-dimension Maslach's Burnout Inventory Scale. Rizzo et al.'s 14-item scale measured role conflict and ambiguity. The government-client working interaction frequency gap was measured by the difference between the five-point level of work interaction frequency with governments minus the one with clients. Structural equation modeling was adopted to test the mediation and moderation models.

**Results:**

Results showed that for the front-line social workers, besides directly reducing personal accomplishment, the government-client work interaction frequency gap could indirectly neutralize its alleviating effects on emotional exhaustion (Mediating effect ratio = −63.64 %), make its total effects on depersonalization significant (37.03%), and reduce personal accomplishment further (23.08%) through increasing social workers' feeling of role conflict. However, the above mediating effects of role conflict were not significant for social workers with extra management or supervision workload, revealing the moderating effects of non-front-line work.

**Conclusions:**

This study revealed that front-line social workers in China who had more work interaction with governments and less with clients could have higher role conflict, increasing their burnout further. Therefore, social work educational programs should include adequate mental adjustment courses and practical emplacement to prepare students for the potential role conflict. Furthermore, higher-level governments should issue relevant regulations to form a collaborative rather than an employment relationship between local governments and social worker organizations.

## Introduction

Since the 2000s, China has witnessed a rapid growth in the number of non-governmental organizations (NGOs), including social work organizations. In 2020, over 669 thousand qualified social workers in China played a vital role in social service and local governance (1). However, social workers have a high risk of burnout, which is considered a worldwide issue (2–5) and concerns the social work industry in China (6, 7). Existing literature associated social workers' burnout with their mental and physical health problems, such as depression (8, 9), sick-leave absences (10), and self-reported health problems (11). The burnout also increased social workers' intentional (12) and actual turnover (13).

Furthermore, when they localize Western social work theories and practices, Chinese social workers find they work in a considerably different environment from their western counterparts as they are highly involved in the local governance (14, 15). To be more specific, compared with the mature social work industry in the west, the professionalism of social work in China is still under development after being abandoned by the revolutionary regime in the 1950s and re-introduced in the late 1980s (16). In addition, Chinese social work tends to be embedded into the existing executive-led and unprofessional social service system (16, 17). The westernized social work ideologies of “individualism, democracy, and Christianity” do not sit comfortably in China's unique social and political backgrounds (18). Therefore, it is crucial to further explore social worker burnout in China's social and political context.

Burnout is characterized as a crisis in one's relationship with work (19). In the condition of burnout, workers become worn out or exhausted as their energy, strength, and resources encounter excessive demands (4). Maslach and Jackson generated a three-dimension definition of burnout, the Maslach Burnout Inventory (MBI), including emotional exhaustion, depersonalization of clients, and reduced personal accomplishment (RPA) (20). These three dimensions were considered to be psychologically discrete (21).

Due to the Chinese government's strong promotion, social work organizations have been playing an important role in social governance in both urban and rural areas (17, 22, 23), but the strong government involvement could be a macro factor causing role stress on social workers (7), which could, in turn, increase their burnout. Role stress is a response that occurs when workers face job requirements that do not match their knowledge and skills, thus challenging their coping ability (24). Role stressor includes two dimensions, i.e., role conflict and role ambiguity (25, 26). Role conflict refers to the feeling of conflicting requirements or competing values at work or requiring the realization of multiple roles in a task (27, 28). Role ambiguity refers to the uncertainty in the definition, expectation, responsibility, and task of one's role, and workers thus do not know what kind of behavior they are expected to do when encountering a specific situation (29). Amplified existing research confirmed the role stress-burnout model that role stress and its two dimensions had a positive direct effect on burnout (30–35), and so had they among social workers (36, 37) and social workers in China (7, 38).

Since the governments contract out massive social services to social work organizations in China, social work organizations' legitimacy and financial resources largely depend on local governments (39, 40). Social workers are thus often in a weaker position when they work with local governments. Therefore, under the contracting-out social services arrangement, they usually have to conduct the additional workload imposed by local governments besides helping their clients with professional skills (41). That administrative workload includes two types: one could be potentially relevant to their professional work, such as assisting government departments with interviewing and investigating, organizing contemporary events, and conducting telephone follow-ups for social service recipients, while the other one is just daily chores utterly irrelevant to their professionalism, such as assisting government departments with sending, receiving or copying documents, drafting unimportant reports, and carrying out reception work (42). If social workers have to deal with local governments too often, they may not have enough time to provide professional services for their clients, and thus the deviation in the time-allocating priority may stress social workers. For example, this additional workload from dealing with governments could cause role ambiguity among social workers as it deviated from their professionalism of focusing on helping clients, especially the underprivileged ones (43, 44). On the other hand, some local governments' demands in China, such as unjustly allocating social welfare resources, emphasizing social control rather than professional caring, and ignoring the underprivileged clients' willingness, conflicted with social work professionalism, and this *anti-social-work* thus could cause role conflict among social workers (45, 46). However, the above viewpoints and findings on the effects of the tension between working with governments and clients on social workers' role stress in China were based on theoretical discussion or qualitative exploration, which few studies have investigated with quantitative methods.

Furthermore, the tension between working with governments and clients reflects the long-standing debate on the contradictory understandings of social work ethics between the conventional western social work view focusing on *helping people to help themselves* and Chinese social work's local knowledge of participating in social governance and conducting administrative affairs (18, 47). The ethics tension could cause burnout among workers (48, 49), and so did the two role stressors through the role stress-burnout model reviewed in the above paragraphs. Therefore, it is necessary to investigate whether the tension between working with governments and clients could impact social workers' burnout through the mediating effects of role ambiguity and conflict, which has not been researched in China.

Existing research indicated that burnout was higher among front-line health care workers than the second-line ones (50, 51). In terms of the social work vocation, the front-line social workers in China could feel the obvious tension between working with governments and clients since they found it was difficult to reconcile the role assigned by local governments with their professional role (45, 52, 53). However, the primary role responsibility of the management of a social work organization was to respond to the funding bodies, usually the local governments in China (54, 55). Therefore, the front-line social workers may experience different role stress caused by the tension between working with governments and clients from the non-front-line ones in China, and such a difference may result in different burnout between those two groups, which little research has investigated.

**Figure 1:**
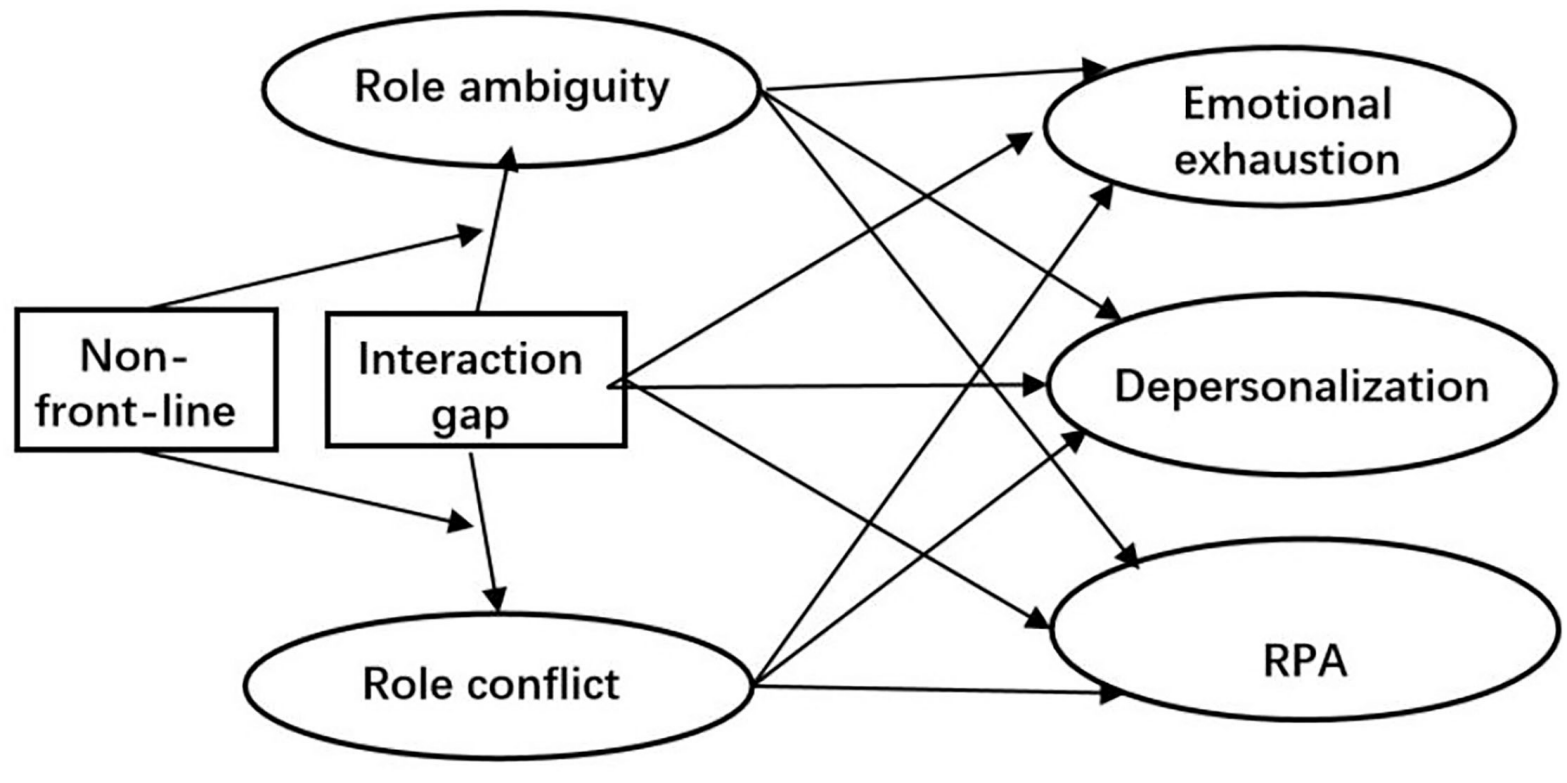
Hypothesized model.

Based on the Role Stress-burnout Model and the previous theoretical and field-work investigations on the tension caused by social workers working with local governments in China, this study examines the effects of the tension between working with governments and clients indicated by the deviation in the social worker's time-allocating priority from their professionalism, which is measured the by the government-client work interaction frequency gap (Interaction gap), on social workers' burnout as well as the mediating effects of role stress (role ambiguity and conflict) and the moderating effects of the non-front-line work (manager and/or supervisor). Understanding how the tension affects social workers' burnout is meaningful, a research gap identified by our literature review. Figure 1 depicts the hypothesized model. For simplicity of the model, this figure only displays the associated paths for the independent, mediating, moderating, and dependent variables, while the path diagram of control variables is omitted.

## Method

### Sample

This study used cross-sectional data from the first wave of the China Social Work Longitudinal Study (CSWLS) in 2019, the first national survey for the social work profession in China. The CSWLS aims to establish a large, comprehensive, and longitudinal dataset about workplace attitudes and behaviors of social workers and the workplace conditions in social work organizations through three rounds of data collection. The first round of data collection for CSWLS was conducted by the East China University of Science and Technology (ECUST) from June to October 2019 [for more details, see Yuan et al.'s introduction (56)].

The CSWLS 2019 received 5,965 individual questionnaires filled in by professional social workers employed by 979 social work organizations located in 56 major cities of 30 province-level administrative regions (except Hong Kong, Taiwan, Macau, and Tibet) all over China. All sampled participants of our study were at least partly involved in front-line social work. After excluding incomplete questionnaires, the sample size is 5,242.

### Ethical Approval

Participants of CSWLS were informed about the aims, objectives, and framework of the survey and assured that their responses would be kept strictly anonymous and confidential. Written informed consent was collected from each participant confidentially. The research ethics committee of ECUST granted the ethical approval for the CSWLS (57). Furthermore, the ethics committee of the first author's university approved the research protocol of this article.

### Measures

#### Dependent Variables

Work burnout was measured by the Chinese version (58) of Maslach's Burnout Inventory-Human Service Survey (MBI-HSS) developed by Maslach et al. (21). The 22-item self-reported MBI-HSS consisted of emotional exhaustion (nine items), depersonalization (five items), and reduced personal accomplishment (RPA, eight items), which three subscales evaluated the emotional reactions caused by excessive work stress, the stress-induced attitudes and feelings toward the service recipient, and the feelings of competence and achievement in one's work separately. The MBI-HSS uses a seven-point Likert-type scale (0 = never and 6 = once every day). Cronbach's alpha for the above three subscales were 0.918, 0.816, and 0.921, respectively.

#### Independent Variable

This study focused on the tension between working with governments and clients indicated by the deviation in the social worker's time-allocating priority from their professionalism. Therefore, we constructed the government-client work interaction frequency gap (Interaction gap) as the independent variable, which was measured by the difference between the self-reported frequency level of work interaction with contract-issuing government departments minus the one with clients. That self-reported work interaction frequency level is a five-point ordinal variable based on the item “How often do you deal with the following types of people (e.g., clients and government departments) at work respectively?” and its responses range from 1 = never to 5 = always. Although the evaluation criteria of interaction frequency level are subjective, the criteria for the governments and the clients are the same for the same person. Therefore, measuring the gap can help reduce the impact of different evaluation criteria between respondents on the estimation.

#### Mediators

The role stress was measured by the Chinese version [as cited in Liao's article (59)] of the shortened form of the Role Conflict and Ambiguity Scales developed by Rizzo et al. (60). The 14-item self-reported scale consists of the nine-item role conflict (RC) and the five-item role ambiguity (RA) sub-scales. The respondents were invited to indicate the degree to which the condition described in the items applied to them on a five-point Likert-type scale ranging from 1 (strongly disagree) to 5 (strongly agree). Cronbach's alpha for the above two subscales were 0.855 and 0.827, respectively.

#### Moderator

We used the variable Non-front-line as the moderator, which was measured as follows: whether the respondent focused on the front-line social work (Non-front-line = 0, front-line social worker, *N* = 2,643) or undertook both front-line social work and supervision or management work (Non-front-line = 1, non-front-line social worker, *N* = 2,599).

#### Controls

This study's controls included three categories of variables: (a) demographic variables of a respondent, including age, gender, and educational attainment, (b) job-related characteristics of a respondent, including monthly income (natural logarithm), length of work experience at the current organization, length of work experience in the social work profession, hours of work per week, and (c) variables of the organization where a respondent worked, including organization age, size of the management board, number of full-time employees, numbers of full-time employees with at least the bachelor's degree, with the social work degree, and with the social work vocational qualification certificate respectively, level of the registered district (province, municipality or district/county), political connection, and the autonomy of expansion (registering a similar organization outside the registered district), service district (providing services outside the registered district), and service content (not undertaking other work arranged by the government outside the contract). We referred to the research of Ye and Sun (40) and Su et al. (57) to design the framework of control variables, and the measurement of those variables can be found in those two articles. We excluded the social support variables and the organization's annual revenue variable due to massive missing values in those variables. Descriptive characteristics of the variables for the front-line and the non-front-line social worker groups are reported separately in Table 1.

**Table 1:** Descriptive statistics of controls for front-line and non-front-line samples.

**Variables**	**Front-line**		**Non-front-line**	
	**Mean/N**	**SD/%**	**Mean/N**	**SD/%**
**Demographic variables**				
Age (17–70)	29.05	7.53	31.45	7.62
Gender				
Male	455	17.22	627	24.12
Female (reference)	2,188	82.78	1972	75.88
Educational attainment				
Junior secondary and below (1)	26	0.98	18	0.69
Senior secondary (2)	218	8.25	121	4.66
Associate's degree (3)	899	34.01	601	23.12
Bachelor's degree (4)	1,371	51.87	1584	60.95
Master's degree and above (5)	129	4.88	275	10.58
Monthly income (CNY)	3275.78	965.13	4349.71	1654.13
**Job-related characteristics**				
Years working in current org. (0–21)	1.61	2.15	3.10	3.05
Social work experience (Year, 0–35)	2.32	2.70	4.62	3.75
Work hours per day (0.2–18)	7.59	1.02	7.90	1.28
**Organization-related variables**				
Organization age (Year)	6.08	3.36	6.20	3.62
Size of the management board (0–27)	5.22	2.67	5.31	2.82
Full-time employees (0–800)	62.96	108.93	73.34	144.59
Full-time Bachelor's degree (0–632)	32.97	68.56	40.33	96.29
employees social work degree (0–216)	20.10	32.45	20.69	35.60
with quali. certificate (0–543)	30.99	58.78	39.11	90.42
Political connection				
Yes	2,081	78.74	2064	79.42
No (reference)	562	21.26	535	20.58
Level of registered district				
Province (reference)	440	16.65	420	16.16
Municipality	780	29.51	803	30.90
County/district	1,423	53.84	1376	52.94
Autonomy of expansion				
Yes	722	27.32	773	29.74
No (reference)	1,921	72.68	1826	70.26
Autonomy of service district				
Yes	1,208	45.71	1189	45.75
No (reference)	1,435	54.29	1410	54.25
Autonomy of service content				
Yes	1,533	58.00	1554	59.79
No (reference)	1,110	42.00	1045	40.21

*Full sample size = 5,242, Front-line sample size = 2,643, Non-front-line sample size = 2,599; 1 CNY ≈ 0.157 USD*.

### Data Analysis

This study adopted structural equation modeling (SEM) to analyze the data and test the model using Stata 16.0 (Stata Corporation, College Station, TX, USA). Since this study has a large sample size, it would not be easy to test the degree of modeling fitness by the χ^2^ (61, 62). We thus used the following three indices that are widely applied to evaluate the model fit: the root mean square error of approximation [RMSEA; (63)], comparative fit index [CFI; (64)], and Tucker–Lewis index [TLI; (65)]. The application of RMSEA, CFI, and TLI is contingent on a set of cutoff criteria: 1) an RMSEA value of <0.05 indicates a close fit, and that <0.08 suggests a reasonable model–data fit (66), 2) a CFI value of > 0.9 indicates that the model is good (64), and 3)TLI >0.90 indicates an acceptable fit (67).

## Results

Pearson's correlation was used to analyze the correlations between the government-client work interaction frequency gap, two role stress variables (i.e., role conflict and role ambiguity), and three burnout variables (i.e., emotional exhaustion, depersonalization, and reduced personal accomplishment) in this study. The means, standard deviations, and correlations for each variable are presented in Table 2 for the sample of front-line social workers and in Table 3 for the sample of non-front-line social workers.

**Table 2:** Means, SDs, and correlations for all variables in SEM of the Front-line sample.

	**Mean ± SD**	**1**	**2**	**3**	**4**	**5**	**6**
Emotional exhau.	12.37 ± 8.84	1.000					
Depersonalization	1.72 ± 0.11	0.523^***^	1.000				
RPA	16.26 ± 9.91	0.150^***^	0.211^***^	1.000			
Role conflict	24.06 ± 5.18	0.344^***^	0.285^***^	0.199^***^	1.000		
Role ambiguity	11.78 ± 2.60	0.214^***^	0.171^***^	0.260^***^	0.166^***^	1.000	
Interaction gap	−1.27 ± 1.21	−0.017	0.058^**^	0.066^***^	0.068^***^	−0.025	1.000

^*^p < 0.05,

** *p < 0.01, and*

*** *p < 0.001.*

**Table 3:** Means, SDs, and correlations for all variables in SEM of the Non-front-line sample.

	**Mean ±SD**	**1**	**2**	**3**	**4**	**5**	**6**
Emotional exhau.	12.37 ± 8.81	1.000					
Depersonalization	1.71 ± 3.12	0.530^***^	1.000				
RPA	15.30 ± 10.00	0.196^***^	0.235^***^	1.000			
Role conflict	24.89 ± 5.60	0.349^***^	0.261^***^	0.219^***^	1.000		
Role ambiguity	11.19 ± 2.74	0.226^***^	0.184^***^	0.298^***^	0.212^***^	1.000	
Interaction gap	−0.65 ± 1.21	−0.012	0.006	0.048^*^	0.035	−0.011	1.000

* *p < 0.05,*

^**^p < 0.01, and

*** *p < 0.001.*

### Test of Measurement Model

The measurement model of five latent variables, including two role stress subscales and three burnout subscales, should be verified before the structural model validation. Results show that the measurement model has a good fit index for the full sample and both the samples of front-line social workers and non-front-line social workers. In terms of the full sample, although χ^2^ (7,275.955, *df* = 545, *N* = 5,242, *p* < 0.001) is significant, the other three indicators show that the model fits well: RMSEA (0.049) is less than the cutoff value of 0.05 while CFI (0.926) and TLI (0.920) are greater than the cutoff value of 0.9. Therefore, RMSEA, CFI, and TLI results show that the measurement model fits well. In addition, the model analysis results show that the selected observation variables effectively reflect the intrinsic structure of the latent variable, as all the variables that make up the latent variable in this model have significant loadings on the latent variable. The standard factor loadings of all the variables that make up the latent variable are between 0.356 and 0.868 (shown in Table 4), and the acceptable factor loading is above 0.3 (68, 69). Therefore, the measurement model confirms the reliability and validity of the latent variables. We also checked the fit index of the measurement model for the front-line sample (RMSEA = 0.049, CFI = 0.925, and TLI = 0.918) and the non-front-line sample (RMSEA = 0.050, CFI=0.924, and TLI = 0.917) separately, and the measurement model fits well with the data of both samples. According to the model analyses for both samples, all the variables' standard factor loadings are above 0.3.

**Table 4:** Standardized factor loadings of observed variables on latent construct.

**Latent construct**	**Observed variables (Items)**	**Factor** **loading**
Emotional	I feel emotionally drained from my work.	0.691
exhaustion	I feel used up at the end of the workday.	0.612
	Working directly with people puts too much stress…	0.674
	I feel I'm working too hard on my job.	0.753
	Working with people all day is really a strain for me.	0.727
	I feel like I'm at the end of my rope.	0.834
	I feel burned out from my work.	0.851
	I feel frustrated by my work.	0.816
	I feel fatigued when I get up in the morning and have…	0.604
Depersonalization	I've become more callous toward people since I took…	0.724
	I worry that this job is hardening me emotionally.	0.718
	I don't really care what happens to some recipients.	0.695
	I feel I treat some recipients as if they were impersonal …	0.657
	I feel recipients blame me for some of their problems.	0.713
RPA	I feel very energetic.	0.694
	I can easily understand how my recipients feel about things.	0.739
	I can easily create a relaxed atmosphere with my recipients.	0.767
	I feel exhilarated after working closely with my recipients.	0.816
	I have accomplished many worthwhile things in this job.	0.855
	I deal very effectively with the problems of my recipients.	0.868
	In my work, I deal with emotional problems very calmly.	0.806
	I feel I'm positively influencing other people's lives …	0.815
Role conflict	I receive an assignment without the proper manpower…	0.500
	I have to buck a rule or policy in order to carry out …	0.455
	I work with two or more groups that operate quite …	0.351
	I receive incompatible requests from two or more people.	0.561
	I do things that are apt to be accepted by one person and…	0.539
	I receive an assignment without adequate resources…	0.639
	I have to work on unnecessary things.	0.695
	Lack of policies and guidelines to help me.	0.725
	I work under incompatible policies and guidelines.	0.676
Role ambiguity	I feel certain about how much authority I have.	0.506
	Clear, planned goals exist for my job.	0.780
	I know that I have divided my time properly.	0.689
	I know what my responsibilities are.	0.833
	I know exactly what is expected of me.	0.615

### Test of Structural Model

**Figure 2:**
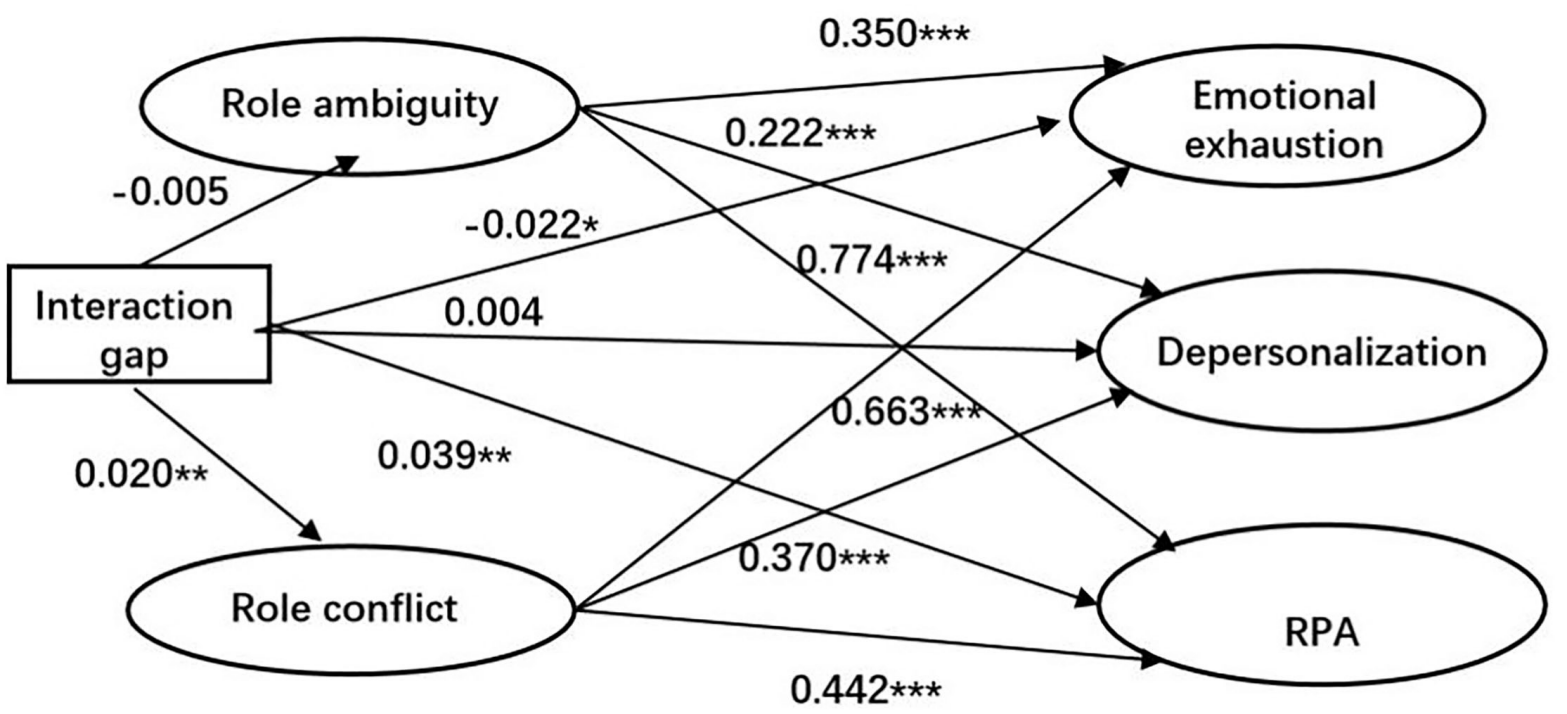
Path coefficient model. *p < 0.05, **p < 0.01, and ***p < 0.001.

We used SEM to test the fitness of the hypothesized model in Figure 1. We first tested the mediation part of the model. Due to the large sample size (*N* = 5,242) and the satisfying results of the other three fitness indicators (RMSEA = 0.037 < 0.05, CFI = 0.914 > 0.9, and TLI = 0.906 > 0.9), the structural model has an adequate fit with the full sample data. Figure 2 displays the standardized results of the associated paths for the independent, dependent, and mediating variables. The diagram shows that the effects of government-client work interaction frequency gap on social workers' burnout are mediated by role conflict (β = 0.020, *p* < 0.01) but not role ambiguity (β = −0.005, *p* > 0.05), which is consistent with the correlation between the Interaction gap and role ambiguity results in Table 2 (β = −0.024, *p* > 0.05) and 3 (β = −0.011, *p* > 0.05). In terms of the mediation effects of role conflict, Tables 2, 3 suggest the potential difference between the samples of front-line and non-front-line social workers as the correlation between the Interaction gap and role conflict is only significant for the front-line social worker sample (β = 0.076, *p* < 0.001). The result indicates that the mediation effects of role conflict are likely to be moderated by the variable of Non-front-line.

Before testing the hypothesized moderation part of the model in Figure 1 by treating the variable of Non-front-line as the moderator, we separately tested the mediation model with the data of the front-line and the non-front-line social worker samples to reveal the direct, indirect and total effects for them. Results shows that the mediation models for both the front-line (RMSEA = 0.036 < 0.05, CFI = 0.917 > 0.9, and TLI = 0.909 > 0.9) and the non-front-line (RMSEA = 0.038 < 0.05, CFI = 0.911 > 0.9, and TLI = 0.903 > 0.9) social worker samples are well fitted. Tables 5, 6 illustrates those two sets of path analysis results respectively.

**Table 5:** Standardized direct, indirect, and total effect and mediating effect ratio for the sample of front-line social workers.

**Endogenous** **Var**	**Path**	**Exogenous** **Var**	**Direct effect** **Coef**	**Indirect effect** **Coef**	**Total effect** **Coef**	**Mediating** **effect ratio**
Role ambiguity	←	Interaction gap	0.008 (0.201)		0.008 (0.201)	
Role conflict	←		0.020 (0.011)		0.020 (0.011)	
Emotional exhau.	←		−0.028 (0.036)	0.016 (0.003)	−0.012 (0.407)	−63.64 %
Depersonalization	←		0.017 (0.095)	0.010 (0.009)	0.027 (0.012)	37.03 %
RPA	←		0.050 (0.004)	0.015 (0.026)	0.065 (0.000)	23.08 %
Emotional exhau.	←	Role ambiguity	0.381 (0.000)		0.381 (0.000)	
Depersonalization	←		0.236 (0.000)		0.236 (0.000)	
RPA	←		0.820 (0.000)		0.820 (0.000)	
Emotional exhau.	←	Role conflict	0.672 (0.000)		0.672 (0.000)	
Depersonalization	←		0.409 (0.000)		0.409 (0.000)	
RPA	←		0.420 (0.000)		0.420 (0.000)	

*P-value is in the parentheses. The word “path” mentioned in the first row of the talbes indicates the meaning of the arrow*.

**Table 6:** Standardized direct, indirect, and total effect and mediating effect ratio for the sample of Non-front-line social workers.

**Endogenous** **Var**	**Path**	**Exogenous** **Var**.	**Direct effect** **Coef**.	**Indirect effect** **Coef**.	**Total effect** **Coef**.	**Mediating** **effect ratio**
Role ambiguity	←	Interaction gap	−0.001 (0.905)		−0.001 (0.905)	
Role conflict	←		0.007 (0.375)		0.007 (0.375)	
Emotional exhau.	←		−0.015 (0.306)	0.005 (0.479)	−0.010 (0.512)	/
Depersonalization	←		−0.005 (0.650)	0.003 (0.483)	−0.002 (0.841)	/
RPA	←		0.052 (0.006)	0.003 (0.683)	0.055 (0.006)	/
Emotional exhau.	←	Role ambiguity	0.318 (0.000)		0.318 (0.000)	
Depersonalization	←		0.189 (0.000)		0.189 (0.000)	
RPA	←		0.724 (0.000)		0.724 (0.000)	
Emotional exhau.	←	Role conflict	0.685 (0.000)		0.685 (0.000)	
Depersonalization	←		0.396 (0.000)		0.396 (0.000)	
RPA	←		0.523 (0.000)		0.523 (0.000)	

*P-value is in the parentheses. The word “path” mentioned in the first row of the tables indicates the meaning of the arrow*.

Table 5 shows that the government-client work interaction frequency gap directly affects two of the three indicators of front-line social workers' burnout. First, it has a significant direct negative influence on the emotional exhaustion of front-line social workers (β = −0.031, *p* < 0.05), indicating that those social workers involved in more work interaction with governments while less with clients have a lower level of emotional exhaustion after controlling for other factors. Meanwhile, the Interaction gap has a distinguished impact on the RPA of front-line social workers (β = 0.048, *p* < 0.01), suggesting that those social workers involved in more work interaction with governments while less with clients have a higher level of reduced personal accomplishment (i.e., lower level of personal accomplishment) after controlling for other factors. However, the effects of Interaction gap on depersonalization are not statistically significant (β = 0.016, *p* > 0.05).

In terms of the indirect influence, the path analysis in Table 5 presents that the Interaction gap has indirect effects on all the three burnout indicators of front-line social workers, whose coefficients are 0.019 (*p* < 0.01) on emotional exhaustion, 0.011 (*p* < 0.01) on depersonalization, and 0.016 (*p* < 0.05) on RPA. The indirect influence mainly takes effect through the mediator, role conflict, rather than role ambiguity. Results suggest that the Interaction gap is significantly associated with higher levels of role conflict (β = 0.024, *p* < 0.01), which, in turn, predicates higher levels of emotional exhaustion, depersonalization, and RPA. However, role ambiguity is not a significant mediator between the Interaction gap and the front-line social workers' burnout.

It should be noted that the directions of the direct and the indirect effects of the interaction gap on emotional exhaustion are opposite, which results in its weak and insignificant total effects (β = −0.012, *p* > 0.05).

Table 6 shows that only the direct and total effects of the Interaction gap on the RPA of non-front-line social workers are statistically significant, while the direct and total effects of the Interaction gap on the other two burnout indicators are not significant.

The results in Table 6 are different from those for the front-line social workers, and especially all the three indirect effects are not significant for the non-front-line social workers. Comparing the results between Tables 5, 6, we found that the association between the Interaction gap and the mediator Role conflict is only significant for the front-line sample (β = 0.024, *p* < 0.01) but not for the non-front-line sample (β = 0.006, *p* > 0.05). Therefore, we further tested whether the mediation effects of the Interaction gap have been moderated by the variable of Non-front-line (vs. Front-line as the reference).

**Figure 3:**
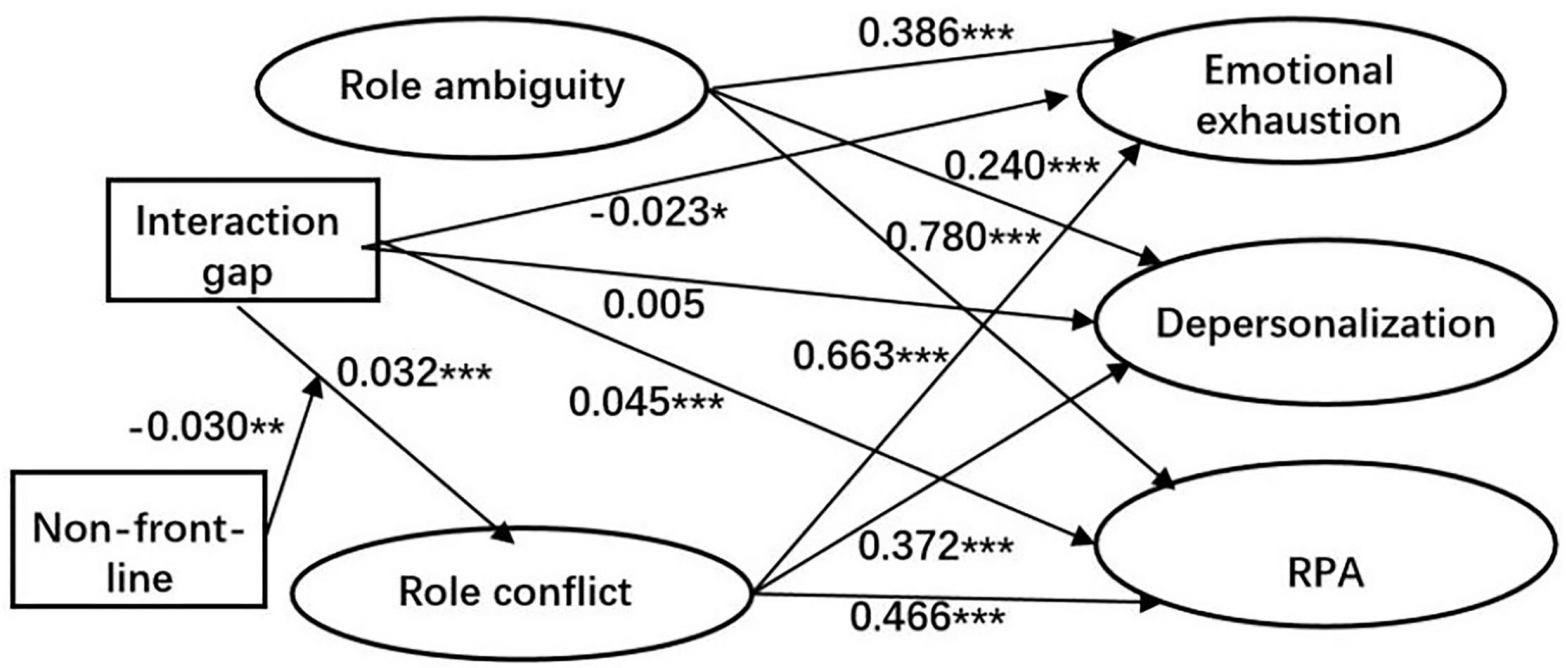
The structural equation model of the moderated mediating effects. *p < 0.05, **p < 0.01, and ***p < 0.001.

According to the above results based on the data of two separate samples, we revised the moderated mediation model by treating the role ambiguity as one control variable rather than one of the mediators. Figure 3 displays the standardized results of the revised model with the data of the full sample. This structural model has an adequate fit (RMSEA = 0.037 < 0.05, CFI = 0.909 > 0.9, and TLI = 0.901 > 0.9). The effects of the interaction item between the Interaction gap and Non-front-line on the association between the Interaction gap and the mediator Role conflict is significant (β = −0.030, *p* < 0.01), which suggests that Non-front-line has moderated the above association.

In terms of the effects of control variables on the three burnout dimensions, the results suggest that: 1) the emotional exhaustion level of social wokers will be higher if they have a higher level of educational attainment (β = 0.039, *p* < 0.05) or more work hours per day (β = 0.046, *p* < 0.001), or work in a older organization (β = 0.010, *p* < 0.024), but be lower if they are older (β = −0.020, *p* < 0.001) or male (β = −0.120, *p* < 0.001); 2) their depersonalization level will be higher if they are male (β = 0.104, *p* < 0.001) but be lower if they are older (β = −0.020, *p* < 0.001); and 3) their RPA level will be lower if they have a higher level of educational attainment (β = −0.065, *p* < 0.01) or work in a organization with the autonomy of providing services outside the registered area (β = −0.130, *p* < 0.001).

## Discussion

This study investigated the effects of the gap between social workers' interaction frequency with governments and clients on their burnout by using the data collected from the CSWLS 2019, the first national survey for the social work profession in China. We also evaluated the mediating effects of role conflict and ambiguity and the moderating effects of non-front-line work. For the non-front-line social workers with extra management or supervision workload, the gap caused by more work interaction with governments and less with clients could only directly reduce social workers' personal accomplishments. However, for the front-line social workers, besides directly reducing personal accomplishment, the government-client work interaction frequency gap could indirectly neutralize its alleviating effects on emotional exhaustion, make its total effects on depersonalization significant, and reduce personal accomplishment further through increasing social workers' role conflict. That is to say, the mediating effects of role conflict are only significant for the front-line social workers but not for social workers with extra management or supervision workload, which reveals the moderating effects of non-front-line work. The findings of this study have implications for social policies and administration guidelines to form a healthy relationship between local governments and social work organizations. They are also an initial basis for psychological intervention and relevant course designs to get social work students and newly graduated social workers well prepared for the workplace.

First, this study establishes that only role conflict mediates the effects of social workers' government-client work interaction frequency gap on their burnout, while role ambiguity does not take effect as a mediator. Consistent with previous studies (32, 33, 35–37), role conflict and ambiguity significantly impact three dimensions of burnout. In addition, this study demonstrates that more work interaction with governments and less with clients is associated with a higher level of role conflict, which quantitative finding is consistent with the theoretical and qualitative investigations of previous research (45, 46). However, the association between the government-client work interaction frequency gap and the social worker's role ambiguity is insignificant. Further study on such a difference is necessary. One possible explanation could be as follows: after more than a decade of vigorous promotion by the government, social workers' participation in local government administrative and other work has been normalized or even standardized. Although the role conflict caused by different work ethics could still be deep-rooted, social workers could have been certain about the definition, expectation, responsibility, and task of working with local governments and clients simultaneously.

Second, this study demonstrates the significant moderating effects of non-front-line work on the association between the social workers' government-client work interaction frequency gap and the mediator of role conflict. Only the front-line social workers' role conflict has indirect positive effects on their burnout. Although the above finding is new and quantitative, it is consistent with previous qualitative findings on the different roles in working with local governments played by the non-front-line social workers from the front-line ones in China (45, 52–55). Since social work supervisors and managers usually have the role of responding to the local governments as the primary funding body in China (55), they could get used to facing conflicting requirements or competing values at work. Thus, the government-client work interaction frequency gap would not significantly result in role conflict among them. However, this explanation requires further empirical investigation.

Third, although front-line social workers' role conflict facilitates the government-client work interaction frequency gap to increase all the three dimensions of their burnout indirectly, we find the Interaction gap is different in its direct effects on those dimensions as follows: 1) significant negative on emotional exhaustion, 2) not significant on depersonalization, and 3) significant positive on reduced personal accomplishment, which results in its different total effects on those three dimensions. Such a difference is consistent with previous academic research on the components of the Maslach Burnout Inventory, whose three dimensions were psychologically discrete and represented different aspects of burnout (21, 38). However, regarding the specific effects of the Interaction gap, it is difficult to compare our results to previous research because there are few quantitative studies on the tension between working with governments and clients on social workers' burnout.

Based on the definition of those three dimensions of burnout (20, 70) and the observations of social workers' work in China (18, 43, 44, 47), we propose possible explanations for its significant adverse effects on emotional exhaustion, which is different from our hypothesis and has a different effect direction from RPA. Previous research observed that social workers often conducted assistant administrative work for local governments (including quasi-government neighborhood committees) besides the professional social work for clients in China (43, 44). Regarding the administrative work commissioned by the government in China, it may have much less emotional demand (e.g., sympathy) from social workers than the “typical” social work services for the clients, most of whom are underprivileged. Thus, the social worker who has more work interaction with governments and less with clients could feel less overextended and depleted of one's emotional resources. However, compared to the typical social work services, which usually help underprivileged children, seniors, and disabled people directly, the administrative work commissioned by governments may give the social workers much less immediate positive feedback. Therefore, the social worker who has more work interaction with governments and less with clients may be more likely to negatively evaluate the achievements at work and feel reduced personal accomplishment.

Regarding the insignificant effects of the Interaction gap on depersonalization, we could consider that long-term administrative work may make those social workers actively ignore the qualities that make the service recipients unique—for example, regarding the recipients as a number in statistics. Such an effect on depersonalization may be no different from the effects of highly stressed professional social work. Further research should investigate the above assumptions.

Several limitations should be acknowledged. First, as only the first wave of the CSWLS was conducted, this study was based on cross-sectional data, which limited our ability to determine causal relationships between independent and dependent variables. The present study's findings should be tested further using the data from the future waves of the CSWLS. Second, the tension between working with governments and clients was not measured directly, and it should include more dimensions related to social workers' investment in work, such as energy and brainpower, besides time. However, due to the data limitation, this paper only investigated the deviation in the time-allocation priority measured by asking social workers' frequency level of work interaction with contract-issuing government departments and the one with clients separately and then calculating the gap between the two levels. More specific direct and multi-dimensional measures of the above tension should be implemented in future research. Third, in order to provide more feasible practical implications, further investigations should be focused on finding some protective factors (e.g., personal resilience, work conditions, and organizational cultures) to mitigate (negative moderate) the effects of the tension between working with governments and clients on role conflict and in turn reduce social worker's burnout. We hope the following waves of the CSWLS or other similar projects can provide relevant data for those studies. Nevertheless, our study provides preliminary but significant evidence to show the vital role of the tension between working with governments and clients in increasing social workers' burnout.

## Conclusion

This study reveals that front-line social workers in China who have more work interaction with contract-issuing government departments and less with clients could have a higher level of role conflict, and the role conflict then could increase their vocational burnout. However, such an association does not apply to social workers with extra management or supervision workload. In order to reduce burnout in social workers, social work educational programs should include adequate mental adjustment courses and practical emplacement to prepare students for the potential role conflict in the workplace. As Chinese social work organizations' reliance on governments will not be significantly changed soon, social work education should prepare students to understand the local knowledge of Chinese social work, which could be different from the knowledge they learned from western textbooks. In addition, front-line social workers should be involved in the management and supervision work as least as an assistant in their early career as soon as possible, which could help them understand the organization's relationship with governments better and thus reduce their role conflict. At the policy level, the professionalization of social work has been promoted significantly in China but still requires more policy support. Local governments should treat social workers as partners rather than clerks, which would benefit social workers' mental health and the government's social governance. Therefore, higher-level governments should issue relevant regulations and administration guidelines on contracting out social services to form a collaborative rather than an employment relationship between local governments and social worker organizations.

## Data Availability Statement

The datasets China Social Work Longitudinal Study 2019 for this study can be acquired by sending the application to the email address of the study's organizer, the East China University of Science and Technology (ECUST): issw@mail.ecust.edu.cn.

## Ethics Statement

The studies (CSWLS) involving human participants were reviewed and approved by the research Ethics Committee of the East China University of Science and Technology that the corresponding author is affiliated with the Ethics Committee of the first author's university Sun Yat-sen University approved the research protocol of this article. The participants provided their written informed consent to participate in this study.

## Author Contributions

JW designed this study and was responsible for data analysis and writing. ZT guided the study design and interpretations, was responsible for data access, and supported the writing. JL supported the data analysis. QW assisted in writing the literature review. All authors approved the final paper.

## Funding

This research was supported by Shanghai Philosophy and Social Science Youth Project (grant no. 2020ESH004).

## Conflict of Interest

The authors declare that the research was conducted in the absence of any commercial or financial relationships that could be construed as a potential conflict of interest.

## Publisher's Note

All claims expressed in this article are solely those of the authors and do not necessarily represent those of their affiliated organizations, or those of the publisher, the editors and the reviewers. Any product that may be evaluated in this article, or claim that may be made by its manufacturer, is not guaranteed or endorsed by the publisher.
